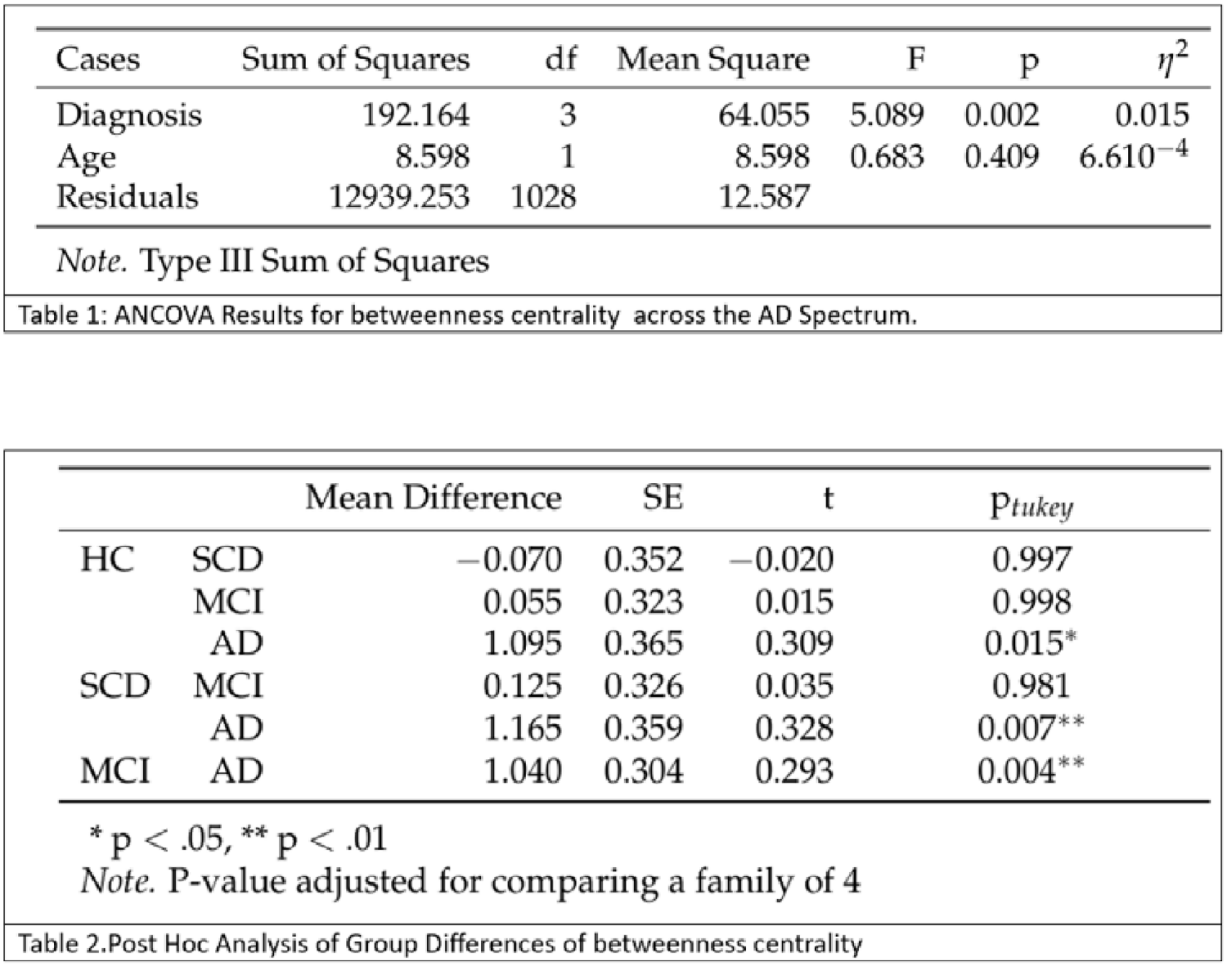# Are Resting‐State EEG Signals the Key to Decoding Functional Brain Networks in Alzheimer's Disease Spectrum?

**DOI:** 10.1002/alz70856_102533

**Published:** 2025-12-25

**Authors:** Vangelis P Oikonomou, Ioulietta Lazarou, Kostas Geordiadis, Spiros Nikolopoulos, Ioannis Kompatsiaris, The PREDICTOM consortium

**Affiliations:** ^1^ Centre for Research & Technology Hellas, Thessaloniki, Greece; ^2^ Stavanger University Hospital, Stavanger, Norway

## Abstract

**Background:**

Alzheimer's disease (AD) is a progressive neurodegenerative condition in which neurons lose their function and connections over time, resulting in deterioration of cognitive abilities. The present study examined the potential of resting EEG signals, under the functional connectivity concept, to discriminate healthy controls (HC) from preclinical stages of AD spectrum using a large cohort of subjects (∼1000).

**Methods:**

We used the CAUEEG dataset which included EEG recordings from 1155 participants. Connectivity metrics provide a robust framework for analyzing brain connectivity. In the present study the betweenness centrality was examined as a network metric. Group‐level differences of betweenness across HC, subjective cognitive decline (SCD), mild cognitive impairment (MCI), and participants with AD were explored using Analysis of Covariance (ANCOVA). Following ANCOVA, pairwise post‐hoc tests were applied.

**Results:**

We provide the statistical analysis of betweenness in alpha band (See Tables 1 and 2). The analysis revealed a significant main effect of diagnosis, F(3, 1031) = 5.089, *p* < .01, indicating that betweenness centrality differed significantly across the groups. Post hoc analyses using Tukey's test were conducted to further examine group differences in betweenness centrality across the AD spectrum. The provided results suggest a progressive decrease in betweenness centrality in alpha band as one moves from HC to SCD, MCI, and then AD, highlighting potential network disruption across the AD spectrum.

**Conclusions:**

This study focuses on leveraging EEG‐based functional connectivity to investigate brain network alterations across the AD spectrum. By employing betweenness centrality we found significant connectivity changes in alpha band of EEGs.

PREDICTOM is supported by the Innovative Health Initiative Joint Undertaking (IHI JU), under Grant Agreement No 101132356. JU receives support from the European Union's Horizon Europe research and innovation programme, COCIR, EFPIA, EuropaBio, MedTechEurope and Vaccines Europe. The UK participants are supported by UKRI Grant No 10083467 (National Institute for Health and Care Excellence), Grant No 10083181 (King's College London), and Grant No 10091560 (University of Exeter). University of Geneva is supported by the Swiss State Secretariat for Education, Research and Innovation Ref No 113152304. See www.ihi.europa.eu for more details.